# Necropsy-Based Analysis of Causes of Neonatal Mortality in Dairy Calves in Poland

**DOI:** 10.3390/ani16111743

**Published:** 2026-06-05

**Authors:** Michal Bednarski, Robert Kupczynski

**Affiliations:** 1 Department of Epizootiology and Clinic of Birds and Exotic Animals, Faculty of Veterinary Medicine, Wroclaw University of Environmental and Life Sciences, 47 Grunwaldzki Sq., 50-366 Wroclaw, Poland; 2 Department of Environmental Hygiene and Animal Welfare, Faculty of Biology and Animal Science, Wroclaw University of Environmental and Life Sciences, 38c Chelmonskiego St., 50-375 Wroclaw, Poland; robert.kupczynski@upwr.edu.pl

**Keywords:** dairy calves, neonatal mortality, diarrhea, septicemia, enteropathogens, colostrum management, necropsy

## Abstract

The aim of this study was to identify the main causes of neonatal mortality in dairy calves (0–30 days of age) in Poland and to assess how infectious factors and management practices influenced the pattern of mortality. A retrospective analysis was performed on 498 calves from 312 herds between 2018 and 2024. The protocol included necropsy, enteropathogen diagnostics (PCR and ELISA), and analysis of herd management questionnaires. The leading causes of death were chronic diarrhea (42.4%), acute diarrhea (25.3%), and septicemia (10.8%). Calves that died from septicemia were the youngest (mean age 7.7 days), whereas those that died from chronic diarrhea were the oldest (20.3 days; *p* < 0.001). Lack of dam vaccination was associated with a higher occurrence of acute diarrhea (RR = 2.04) and septicemia (RR = 2.01) within the necropsied calf population. Inappropriate colostrum management was also associated with a higher occurrence of septicemia (RR = 1.99). Despite widespread antimicrobial use (>80%), intensive fluid therapy was used rarely (<7% in diarrheal cases). ETEC, rotavirus, and coronavirus were detected significantly more frequently in acute diarrhea, whereas *Cryptosporidium parvum* (*C. parvum*) was significantly more frequent in chronic diarrhea. Antimicrobials were used extensively, while intensive fluid therapy for diarrhea was implemented infrequently and inadequately relative to disease severity. This study makes an important contribution to the understanding of calf mortality in dairy herds by combining comprehensive postmortem diagnostics with pathogen detection (PCR and ELISA) and the assessment of management practices, thereby providing a holistic perspective on the problem. The analysis of 498 calves from 312 farms over a 7-year period offers an unprecedented and representative picture of the situation in Poland, one of Europe’s major milk producers. These results emphasize the crucial importance of proper colostrum management and vaccination, while also indicating a clear and urgent need to expand the use of fluid therapy in calves requiring treatment. The results identify not only the dominant pathogens and differences in disease course, but also management factors with direct implications for animal welfare and preventive strategies in dairy production.

## Simple Summary

Calf deaths in the first month of life are a major problem on dairy farms, affecting both animal welfare and farm profitability. This study analyzed nearly 500 cases from over 300 farms in Poland to identify the main causes of death. The most common cause of death was diarrhea, followed by infections, with the youngest calves most at risk of severe infections. Higher mortality was linked to lack of vaccination of cows and poor management of colostrum, the first milk that provides essential immunity to newborn calves. Although antibiotics were widely used, proper fluid treatment for dehydration was rarely applied. The results show that improving early calf care, especially colostrum feeding and cow vaccination, along with better use of fluid therapy, could significantly reduce calf mortality.

## 1. Introduction

Proper calf rearing requires a holistic approach that integrates nutritional, environmental, health-related, and organizational factors. Calf health and survival during the first weeks of life result from the complex interaction of herd management, periparturient care, environmental conditions, and infectious pressure [1,2,3,4,5]. Neonatal calf mortality is most commonly defined as the number or proportion of live-born calves that die within the first 28–30 days of life. The most common direct causes of neonatal calf death include gastrointestinal infections, respiratory infections, abomasal disorders, umbilical infections, and congenital defects. Their occurrence is further shaped by environmental and management-related factors such as hygiene, nutrition, housing conditions, and calf-rearing organization [1,3,4,6,7,8]. It is also well established that management practices, particularly appropriate colostrum management, can reduce calf mortality at the herd level [6,8,9,10]. Studies have shown that failure of passive transfer is associated with an increased risk of diarrhea and death during the first month of life [7,8,11,12].

In dairy calves, neonatal mortality usually ranges from 2% to 7%, although reported values vary considerably between studies [9,11]. A study of 93 farms in Germany found a mean calf mortality rate of 7.3% during the first 6 months of life, with substantial between-herd variation [8]. Other studies suggest that increasing herd size may be associated with calf mortality exceeding 10% in some herds [10]. However, comparisons between studies are complicated by differences in methodology, particularly by variation in the age thresholds used to define neonatal mortality [6].

Poland is an important milk producer in Europe, and the domestic dairy sector is characterized by considerable structural diversity. Milk production is carried out both in small family farms and in large, specialized operations with a high degree of technological advancement [13]. In recent years, a steady increase in the average number of cows per herd and the expansion of large farms have been observed; these holdings generally have better access to modern technologies, higher standards of animal welfare, and more stringent hygiene protocols [13]. This diversity may substantially affect disease incidence, mortality, and the distribution of causes of calf loss [14], which justifies the need to analyze the determinants of neonatal calf mortality under Polish conditions. It is estimated that approximately 30% of dairy calves are treated for at least one disease episode before 6 months of age [11]. Therefore, the implementation of early detection systems and well-designed monitoring, diagnostic, and treatment protocols is essential to improve therapeutic outcomes and reduce losses [1,15].

The aim of the present study was to determine the causes of calf mortality during the first month of life on the basis of postmortem examinations, case histories, and herd management data. Particular attention was paid to infectious factors, including enterotoxigenic *Escherichia coli* (ETEC), rotavirus, bovine coronavirus, and *Cryptosporidium parvum* (*C. parvum*). We hypothesized that a holistic diagnostic approach would provide data enabling a better understanding of the factors determining neonatal mortality in dairy calves.

## 2. Materials and Methods

### 2.1. Study Design and Case Selection

This retrospective study was designed as a necropsy-based case series in dairy calves during the neonatal period. The analysis included 498 calves that died within the first 30 days of life and were referred for postmortem examination between 2018 and 2024. The animals originated from 312 commercial dairy herds in Poland. Additional cases from the same farm were included only if they occurred in a subsequent calendar year, in order to limit overrepresentation of individual herds. Stillbirths and deaths directly related to severe dystocia, perinatal trauma, or asphyxia were excluded so that the final material reflected mortality during the neonatal period [4,16,17]. Because submission for necropsy depended on the decisions of the attending veterinarians and herd owners, the dataset represents a referral-based diagnostic case series. As such, it was used to describe and classify the causes of death in this necropsy-base study [18].

### 2.2. Herd- and Calf-Level Data

For each calf, herd-level information was collected from submission forms, veterinary records, and herd interviews. The general structure of the questionnaire and the issues it covered are provided in the Supplementary Material (Table S1.1). Ethical approval for this study was granted by the Research Ethics Committee of the Wroclaw University of Environmental and Life Sciences (Resolution No. NON00000.0020). Herds were classified according to the number of cows as small (20–49), medium (50–199), or large (≥200). Variables included calf age at death, season, dam vaccination status (vaccine against *E. coli* F5, rota-, coronavirus), colostrum management, water availability, and treatments administered, including fluid therapy and antimicrobial therapy. Documented appropriate colostrum management was defined as fulfillment of two criteria simultaneously: administration of an adequate quantity of colostrum within the appropriate time after birth and assessment of colostrum quality using the Brix scale, (≥22%). Because laboratory indicators of passive immunity transfer were not available for all calves, this variable was not interpreted as confirmed successful transfer of passive immunity, but rather as an indicator of appropriate husbandry practices during the first hours of life [12].

### 2.3. Necropsy Examination and Diagnostic Classification

All calves underwent complete necropsy, including external examination and systematic inspection of the internal organs. Gross pathological findings, herd history, and laboratory results were analyzed jointly, and each case was subsequently assigned to a diagnostic category. If more than one disease process was identified in a single calf, the main cause of death was defined as the factor most likely to have initiated the sequence of changes leading to death, whereas the remaining processes were recorded as concurrent findings [18]. Concurrent findings, such as secondary septicemia, pneumonia, omphalitis, abomasal lesions, or wasting, were recorded but were not used to create overlapping primary outcome categories.

Acute diarrhea was diagnosed when diarrheal signs had been present for up to 3 days before death and necropsy findings indicated an acute gastrointestinal process, without marked body wasting or evidence of prolonged nutritional deterioration. Chronic diarrhea was diagnosed when diarrheal signs had persisted for more than 3 days and were accompanied by postmortem evidence of prolonged gastrointestinal dysfunction, chronic dehydration, progressive loss of body condition, emaciation, or secondary digestive disturbances. The 3-day threshold was used as an operational retrospective criterion to distinguish short, rapidly fatal enteric disease from prolonged diarrheal disease with systemic and nutritional consequences. Because definitions of neonatal calf diarrhea differ substantially among published studies, this classification was intended to provide a transparent and reproducible case definition within the present dataset [14,19,20].

Septicemia was diagnosed when generalized infection was confirmed by postmortem findings and by bacteriological isolation of the same bacterial species from three or more sampled organs. Such postmortem findings included different signs such as petechiae, fibrin, sometimes accompanied to free fluid in body cavities. The wasting syndrome category included calves with marked emaciation associated with chronic disease, prolonged undernutrition, or chronic gastrointestinal dysfunction, regardless of whether septicemia was also present as a secondary condition. All remaining primary diagnoses were classified as other causes [18]. Hemorrhagic enteritis, emphysematous enteritis, and intussusception were grouped within a single category termed “enteric disorders”.

### 2.4. Bacteriological Culture and Enteropathogen Detection

Routine aerobic bacteriological culture was performed at 37 °C for 24 h on Columbia agar supplemented with 5% sheep blood, MacConkey agar, and Sabouraud dextrose agar (SDA) (bioMérieux, Marcy-l’Étoile, France). Samples from the liver, spleen, lungs, and intestines were collected from each calf for bacteriological examination. For the detection of *Salmonella* spp., samples were pre-enriched in Buffered Peptone Water, enriched in Rappaport–Vassiliadis broth, and subsequently cultured on Xylose Lysine Deoxycholate agar (XLD) and Salmonella-Shigella (SS) agar (bioMérieux, Marcy-l’Étoile, France). Biochemical identification and serotyping were then performed according to the laboratory’s routine procedure compliant with EN ISO 6579-1:2017 [21].

Additional anaerobic and microaerophilic cultures were not performed routinely in all calves but were limited to selected cases in which gross postmortem findings suggested the possible involvement of anaerobic or microaerophilic bacteria. Such testing was applied particularly in cases of necrotizing, hemorrhagic, or emphysematous enteritis, abomasitis, and necrotic lesions in internal organs. Anaerobic culture was performed on Columbia agar supplemented with 5% sheep blood and in Schaedler broth with vitamin K3 (bioMérieux, Marcy-l’Étoile, France) and incubated at 37 °C for 48–72 h under anaerobic conditions using the GENbag anaer system (Oxoid, Basingstoke, UK). Microaerophilic culture was performed on Columbia agar supplemented with 5% sheep blood and incubated at 37 °C for 48–72 h under microaerophilic conditions using the GENbag microaer system (Oxoid, Basingstoke, UK).

In calves classified into the diarrhea category, additional tests were performed to determine the infectious etiology, including PCR-based identification of *E. coli* strains belonging to the ETEC was defined based on the detection of virulence factors consistent with the ETEC profile (F5, F41, F5/F41, F17, and EAST1) using PCR (Thermo Fisher Scientific, Waltham, MA, USA) as previously described by Bertin et al. [22], Casey and Bosworth [23], and Bednarski [24]. For each case, five morphologically typical *E. coli* colonies were tested to reduce the risk of missing ETEC, no case showed the presence of more than one pathotype. Detection of rotavirus, bovine coronavirus, and *C. parvum* was performed using commercial assays (Bio-X Diagnostics, Rochefort, Belgium). To ensure consistency with the diagnostic classification applied in this study, diarheic calves assigned to the acute diarrhea and chronic diarrhea categories.

### 2.5. Statistical Analysis

Statistical analyses were performed using the Statistica software package (ver. 13.3, Software Inc., New York, NY, USA). Before performing the actual analyses, the assumptions of the statistical model were verified. Continuous variables were summarized descriptively as mean ± standard deviation (SD), whereas categorical variables were expressed as counts and percentages. The normality of continuous data distribution was evaluated using the Shapiro–Wilk test. Because the distribution of age at death did not satisfy the assumption of normality, comparisons of age among diagnostic categories were conducted using the Kruskal–Wallis rank-sum test. The primary outcome variable was cause-of-death category, classified as acute diarrhea, chronic diarrhea, septicemia, cachexia/wasting syndrome, or other causes. Descriptive distributions of diagnostic categories were first summarized for the entire dataset and then stratified according to season and herd-size category. Season was derived from calendar month and classified as winter (December–February), spring (March–May), summer (June–August), and autumn (September–November). Associations between categorical explanatory variables and the distribution of diagnostic categories were assessed using Pearson’s chi-square test. This approach was used for the overall comparisons involving herd size, vaccination status, colostrum management, and water access categories. For the supplementary pairwise analyses of selected management-related exposures (non-vaccinated vs. vaccinated; improper vs. proper colostrum management; periodic vs. adequate water access; no water access vs. adequate water access; oral rehydration therapy vs. no fluid therapy; intravenous/subcutaneous fluid therapy vs. no fluid therapy; antibiotic therapy: yes vs. no), each diagnostic category was additionally modeled as a binary endpoint (presence of the category of interest = 1; all remaining causes of death = 0).

To evaluate the effects of management factors (vaccination, colostrum administration, and water access) and pathogen detection on the pattern of calf mortality, relative risk (RR) and odds ratio (OR) estimates were calculated. RR and OR were reported with 95% confidence intervals calculated using the Wald method on the logarithmic scale. An RR > 1 indicated an increased risk of death from a given cause in the exposed group. Because the study was based on calves submitted for necropsy and did not include population-at-risk denominators for all live-born calves in the source herds, RR estimates were interpreted only as descriptive comparative measures within the necropsied study population. OR was defined as the ratio of the odds of occurrence of a given disease entity. Statistical significance was set at *p* < 0.05; results with higher confidence were denoted as *p* < 0.01 and *p* < 0.001.

## 3. Results

### 3.1. Main Causes of Death and Age at Death

Analysis of the main causes of death showed clear variation in frequency according to diagnostic category (Table 1). Necropsy findings in 498 calves indicated that chronic diarrhea was the most common cause of death (42.4% of all cases), followed by acute diarrhea, septicemia, and cachexia/wasting syndrome and other causes. The “other” category comprised a heterogeneous group of less frequent causes of death, including primarily abomasal disorders, namely severe abomasal ulceration (n = 22, 4.4%), abomasal ulceration with peritonitis (n = 13, 2.6%), and abomasitis (n = 3, 0.6%); enteric disorders (n = 8, 1.6%) and emphysematous enteritis (n = 7, 1.4%); respiratory diseases, including pneumonia (n = 7, 1.4%) and aspiration pneumonia (n = 4, 0.8%); and other causes. The mean age of calves that died from chronic diarrhea was 20.27 ± 5.56 days, compared with 10.78 ± 5.16 days for acute diarrhea and 7.65 ± 4.96 days for septicemia. Differences in age among causes of death were statistically significant (*p* < 0.001).

### 3.2. Seasonal Distribution of the Main Causes of Death

Deaths caused by acute diarrhea also showed a clear seasonal pattern. The highest proportion of cases occurred in winter (39.7%) and spring (37.3%), whereas the fewest deaths were recorded in summer (Table 2). Analysis of calf mortality revealed an association between season and cause of death. Chronic diarrhea, the most common cause of death, occurred throughout the year, but the highest proportion of cases was recorded in winter (31.3%), followed by autumn (28.0%). Lower values were observed in spring and summer. Septicemia showed a bimodal seasonal distribution, with the highest values observed in winter and autumn (33.3% each). The lowest proportion of cases was recorded in summer. In wasting syndrome, the highest proportion of deaths occurred in autumn (33.3%) and spring (29.6%), whereas winter and summer values were lower. Deaths classified as other causes were recorded most often in spring (30.0%), followed by winter (28.8%) and autumn (27.5%), with the lowest proportion occurring in summer (13.8%).

### 3.3. Association Between Farm Size and Diagnostic Categories

Analysis of herd-size distribution showed that most cases originated from large herds. A significant association was found between farm size and the distribution of causes of death (χ^2^ = 28.76). Chronic diarrhea predominated across all farm-size categories, but medium-sized farms showed relatively higher proportions of septicemia and cachexia/wasting syndrome than large and small farms (Table 3).

An exploratory comparison of season distribution by farm size did not reach statistical significance (χ^2^ = 9.68, df = 6, *p* = 0.139) and should therefore be interpreted cautiously. Given the absence of population denominators, this comparison is descriptive only.

### 3.4. Association Between Management Factors and Diagnostic Categories

The distribution of risk factors differed substantially between disease categories (Table 4), with the strongest association observed for vaccination status. Among vaccinated calves, mortality was most frequent in the chronic diarrhea group (69.7%), whereas among calves with acute diarrhea and septicemia, unvaccinated animals predominated (65.1% and 64.8%, respectively). In the categories of other causes and cachexia/wasting syndrome, the proportions of none-vaccinated calves were 55.0% and 48.1%, respectively. Appropriate colostrum management was most often observed in chronic diarrhea cases (54.0%), whereas inappropriate colostrum management predominated in septicemia and wasting (70.4% each), as well as in other causes (60.0%). Appropriate colostrum management was recorded in 45.2% of calves with acute diarrhea, whereas improper colostrum management was recorded in 54.8%. Adequate water access predominated in most causes of death, reaching 58.8% in chronic diarrhea, 66.7% in acute diarrhea, and 62.5% in the other-causes group. In septicemia and wasting, intermittent water access was relatively more common (48.1% and 55.6%, respectively).

Detailed analyses of individual risk factors are presented in Supplementary Table S1.2. Lack of vaccination was significantly associated with the distribution of selected diagnostic categories. Non-vaccinated calves showed a markedly higher risk of acute diarrhea and septicemia, with RR values of 2.04 (95% CI: 1.48–2.81) and 2.01 (95% CI: 1.18–3.42), respectively, whereas the risk of chronic diarrhea was significantly lower in this group (RR = 0.48, 95% CI: 0.38–0.60). No significant associations were found for cachexia/wasting syndrome or other causes of death. Improper colostrum management was significantly associated with a higher occurrence of septicemia and a lower occurrence of chronic diarrhea. Calves with improper colostrum intake had an almost twofold higher risk of septicemia (RR = 1.99, 95% CI: 1.14–3.47), while the risk of chronic diarrhea was significantly reduced (RR = 0.71, 95% CI: 0.58–0.88). No statistically significant associations were observed for acute diarrhea, cachexia/wasting syndrome, or other causes. Periodic access to water was significantly associated with the occurrence of several diagnostic categories when compared with adequate water access. In this group, the risk of septicemia (RR = 1.74, 95% CI: 1.04–2.92) and cachexia/wasting syndrome (RR = 2.10, 95% CI: 1.00–4.37) was higher, whereas the risk of acute diarrhea was significantly lower (RR = 0.66, 95% CI: 0.46–0.94). No significant associations were identified for chronic diarrhea or other causes of death. Complete lack of water access was not significantly associated with any of the evaluated diagnostic categories. Although point estimates suggested some variation in risk across categories, all confidence intervals were wide and all *p*-values remained above 0.05. These findings indicate that, in contrast to intermittent water access, the “no access” category did not show statistically robust associations in this dataset.

### 3.5. Enteropathogen Detection in Acute and Chronic Diarrhea

ETEC, rotavirus, and coronavirus were detected significantly more frequently in acute diarrhea than in chronic diarrhea cases (*p* < 0.01). The strongest association with acute diarrhea was observed for ETEC (51.6%) and rotavirus (33.3%). Coronavirus was also markedly more common in acute diarrhea (23.0% vs. 0.5%). In contrast, *C. parvum* was detected more frequently in chronic diarrhea cases (37.4% vs. 25.4%). *Salmonella* spp. was detected only sporadically and showed no significant association with diarrhea type (Table 5). Mixed infections involving bacterial, viral, and protozoal pathogens were also observed in some calves. Mixed infections were more common in acute than in chronic diarrhea (38 vs. 9 cases). The most frequent pattern was the simultaneous detection of ETEC, rotavirus, bovine coronavirus, and *C. parvum* (n = 12), observed only in acute diarrhea. Coinfections involving ETEC and rotavirus predominated in acute cases, whereas combinations with *C. parvum* were relatively more frequent in chronic diarrhea (Supplementary Table S1.3).

### 3.6. Seasonal Distribution of Pathogen Detection in Diarrheic Calves

The occurrence of enteropathogens varied by season (Table 6). None of the evaluated pathogens showed statistically significant seasonal differences (*p* > 0.05). ETEC was detected most often in winter (37.6%) and spring (27.1%) and less often in summer (16.5%) and autumn (18.8%), but this difference was not significant (χ^2^ = 1.56; *p* < 0.669). Rotavirus and *C. parvum* showed broadly similar seasonal distributions, despite slightly higher detection rates in winter and spring, but these differences did not reach statistical significance (*p* > 0.05). Coronavirus was identified most often in spring (43.3%) and winter (36.7%) and least often in autumn (6.7%), showing a non-significant trend toward seasonality (χ^2^ = 6.17; *p* < 0.103). Detection of *Salmonella* spp. remained low in all seasons and was not associated with season (*p* < 0.349).

### 3.7. Pre-Mortem Supportive Treatment and Antibiotic Use

Supportive treatment data are presented descriptively. No fluid therapy was recorded most often in calves with septicemia and other causes, whereas oral rehydration therapy was most frequent in acute diarrhea (Table 7). Intravenous/subcutaneous fluid therapy was used only rarely. Antibiotic therapy was common across all diagnostic categories.

The associations between selected therapy-related factors and cause-of-death categories are presented in Supplementary Table S1.4. Supportive fluid therapy differed significantly across causes of death (*p* < 0.001), whereas antibiotic use did not vary significantly between diagnostic categories (*p* = 0.304). Oral rehydration therapy was strongly associated with acute diarrhea (RR = 2.81, 95% CI: 2.08–3.81; OR = 4.31, 95% CI: 2.77–6.70) and occurred less frequently in septicemia and other causes of death. Intravenous/subcutaneous fluid therapy was also more frequent in acute diarrhea (RR = 2.76, 95% CI: 1.56–4.87), while no statistically supported associations were identified between antibiotic therapy and any individual cause-of-death category (Supplementary Table S1.4).

## 4. Discussion

The neonatal period is characterized by the highest susceptibility to disease and death, owing to intense environmental pressure and exposure to enteropathogens typical of the first weeks of life, such as *Escherichia coli* strains, rotavirus, coronavirus, and *Cryptosporidium* spp. [3,4,9,20,25]. In the present study, diarrheal syndromes were the predominant cause of death during the first month of life, together accounting for 67.7% of cases, with chronic diarrhea representing 42.4% and acute diarrhea 25.3% of all diagnoses. This pattern is consistent with findings from other neonatal populations in which gastrointestinal disease is a leading contributor to morbidity and mortality in calves. Mee [3] identified gastrointestinal infections as the most common disease group responsible for neonatal calf mortality and estimated the incidence of diarrhea during this period at approximately 20–40%, with mortality reaching 8%. Cho and Yoon [25], citing NAHMS data, reported that diarrhea accounted for 57% of preweaning calf mortality in US dairy herds, with most cases occurring in calves younger than 1 month; they also cited data from Korea, where diarrhea accounted for 53.4% of deaths in dairy calves. In a Uruguayan herd-level study, the annual risk of calf mortality from birth to weaning was 15.2%, and diarrhea was the most frequently reported clinical syndrome preceding death, occurring on 85.2% of farms, whereas respiratory disease was reported on 47.5% of farms [2]. In contrast, respiratory disease predominated in commercial calf ranches in the United States, accounting for 67.5% of necropsy cases, whereas gastrointestinal disorders accounted for 11.5% and septicemia for 9.5% [26]. This suggests that the predominance of diarrheal syndromes in our material is more typical of strictly neonatal dairy calf populations than of systems based on the transport and commingling of animals from multiple sources, where respiratory disease pressure is greater. From this perspective, our findings seem most comparable to studies conducted in calves during the first 2–4 weeks of life rather than to material derived from later stages of rearing. Particularly noteworthy is the very high proportion of chronic diarrhea.

Calf diarrhea is categorized according to clinical signs, duration, and etiological factor. Wilson et al. [27] showed that the literature uses highly variable definitions of neonatal calf diarrhea and different diarrhea-related endpoints, which substantially limits direct comparison across studies. In this context, the high proportion of chronic diarrhea in our material should be interpreted primarily as a consequence of more detailed postmortem classification. This interpretation is further supported by cohort studies. Schinwald et al. [28] reported that calves experienced diarrhea during an average of 16% of the first 28 days of observation, and that an increasing proportion of days with abnormal fecal consistency increased the risk of death and reduced weight gain. Goh et al. [29] likewise confirmed that diarrhea during the preweaning period is an important cause of calf mortality and may also have longer-term health and production consequences. In light of these data, the predominance of chronic diarrhea in our study suggests that a substantial proportion of mortality was due to uninterrupted disease progression and its metabolic consequences rather than to a single acute, short-lived enteric episode. In the present study, omphalitis was not identified during postmortem examinations. However, other studies have shown that risk factors for omphalitis include sex, umbilical cord length, and birth weight, as well as farm-related factors such as bedding moisture and inadequate umbilical disinfection [30].

In our study, calves dying from septicemia were the youngest, with a mean age of 7.7 ± 5.0 days, whereas calves dying from chronic diarrhea and wasting syndrome were clearly older. This pattern is consistent with the classical course of neonatal disease, in which generalized infections occur earliest, during the period when the neonate is most dependent on effective passive immunity transfer and the quality of postpartum care, whereas the chronic consequences of diarrhea become evident later, after several or more days of illness [3,4]. In the study by Lora et al. [7], failure of passive transfer increased the odds of disease 24-fold and the odds of death 11-fold in calves younger than 30 days and was also associated with an earlier onset of illness. This relationship is consistent with our findings, in which inappropriate colostrum management was most common among calves that died of septicemia and wasting.

In the present study, acute diarrhea contributed more often to deaths in winter and spring, whereas chronic diarrhea remained the dominant category throughout the year. Raboisson et al. [9] reported that calf mortality during the first month of life in France reached 12–17% in winter and was lower in summer (8–12%). In Norwegian herds, calves born in winter were more likely to die during the first week and during the first month of life than calves born in summer or autumn, and enteritis was among the most common postmortem diagnoses [6]. In the study by Berber et al. [19], admissions of calves with severe diarrhea to the ICU and pathogen detection rates were highest in winter. Our findings therefore suggest that, under Polish conditions as well, the cold season may predispose calves to a more severe course of gastrointestinal disease. By comparison, Jurkovich et al. [31] reported that daily mortality in hutch-reared dairy calves was higher in summer and winter than in spring and autumn, indicating that thermal stress at both ends of the temperature spectrum may impair calf survival. However, direct comparison should be made with caution because their study evaluated overall mortality in preweaned calves on a single farm, whereas our study was based on necropsy-classified neonatal deaths and therefore reflects cause-specific fatal outcomes rather than total on-farm mortality.

The relationship between herd size and the distribution of causes of death should also be interpreted cautiously, but it should not be overlooked [3,4,32,33]. In our material, chronic diarrhea predominated across all herd-size categories, whereas septicemia and wasting syndrome were relatively more common in medium-sized herds. In Norway, mortality increased with herd size, and calves housed in groups from the second week of life had a higher risk of death during the first month [6]. An Irish analysis likewise showed that increasing herd size significantly affected calf mortality, which may reflect management challenges associated with herd expansion [10]. Our findings indirectly suggest that management-related challenges associated with increasing herd size may contribute to the observed differences in causes of calf death.

Among the necropsied calves, a significant association was found between the cause of death and colostrum management. Inappropriate colostrum management was recorded in 70.4% of calves that died of septicemia and in 70.4% of calves with wasting syndrome, whereas the corresponding proportion in chronic diarrhea was 46.0%. These findings are biologically plausible. Lora et al. [7] showed that failure of passive transfer increased the odds of disease 24-fold and death 11-fold and was also associated with a higher risk of rotavirus infection and cryptosporidiosis. In the study by Keller et al. [8], which included 93 large herds and 54,474 live-born calves, lower mortality was observed on farms where calves received colostrum directly from the dam. Consequently, our results reinforce the conclusion that a proportion of mortality later attributed to diarrhea, septicemia, or wasting has its origin in errors made during the first hours of life. In our material, calves classified as chronic diarrhea more often came from vaccinated dams, whereas unvaccinated status predominated in acute diarrhea and septicemia. Other studies indicate that a properly implemented vaccination program against rotavirus, coronavirus, and ETEC F5 can reduce the hazard of diarrhea-related mortality. Viidu and Mõtus [34] showed that, in herds adhering to vaccination recommendations and feeding transition milk for at least 14 days, the hazard of death due to diarrhea decreased to an average of HR = 0.72, and in herds that vaccinated appropriately but fed transition milk for a shorter period, even to HR = 0.24; conversely, in herds deviating from recommendations, the hazard increased to HR = 1.61. In practice, this means that vaccination should be regarded as one component of a broader preventive package, the effectiveness of which depends on the quality of the colostrum program and overall neonatal management rather than as a stand-alone solution. In our study lack of dam vaccination was associated with a higher occurrence of acute diarrhea (RR = 2.04) and septicemia (RR = 2.01) within the necropsied calf population. Inappropriate colostrum management was also associated with a higher occurrence of septicemia (RR = 1.99). RR estimates were interpreted only as descriptive comparative measures within the necropsied study population. Therefore, they should not be regarded as estimates of true disease incidence or mortality risk in the general dairy calf population.

The distribution of infectious agents identified in our study showed clear differences between acute and chronic diarrhea. In acute diarrhea, ETEC was detected in 51.6% of cases, rotavirus in 33.3%, and coronavirus in 23.0%, whereas in chronic diarrhea the corresponding proportions were 9.5%, 3.3%, and 0.5%, respectively. In contrast, *C. parvum* was recorded more frequently in chronic than in acute diarrhea (37.4% vs. 25.4%, *p* < 0.02). This pattern is consistent with the classical etiological model in which ETEC, rotavirus, and BCoV are among the principal causes of acute diarrhea in the youngest calves, while cryptosporidiosis is more often associated with a more prolonged clinical course and longer-lasting intestinal damage [25,33]. Previous studies have shown that at least one pathogen can be detected in 65.4% of calves with diarrhea [34]. In the study by Berber et al. [19], which included hospitalized calves with severe diarrhea, *Cryptosporidium* spp. (61.5%) and rotavirus (56.4%) were detected most frequently, and mixed infections were common. Likewise, the meta-analysis by Brunauer et al. [20] demonstrated that among mixed infections, the highest pooled prevalence was found for the rotavirus and *Cryptosporidium* combination (6.69%), followed by rotavirus and coronavirus (2.84%), rotavirus and ETEC (1.64%). Our findings indicate that neonatal diarrhea was multifactorial in origin, and that the final clinical presentation depended on calf age, disease duration, and the contribution of mixed infections.

Our findings revealed a clear imbalance between the frequency of antimicrobial use and the use of intensive fluid therapy. Antimicrobials were administered to 89.7% of calves with acute diarrhea, 87.7% of those with chronic diarrhea, and 85.2% of those with septicemia, whereas intravenous or subcutaneous fluid therapy was used in only 6.3% of calves with acute diarrhea and 4.7% of those with chronic diarrhea. No fluid therapy was recorded in calves with septicemia. This pattern should be interpreted cautiously because the retrospective records did not include treatment timing, initial dehydration severity, clinical progression, antimicrobial class, dose, duration, or susceptibility testing.

Diarrhea remains the leading cause of calf mortality, and oral electrolyte therapy is a simple method of counteracting dehydration, acidosis, and negative energy balance [35,36]. Berchtold [37] emphasized that severely dehydrated calves unable to suckle require intravenous fluid therapy. Constable [35], in turn, recommended supportive treatment and antimicrobial therapy mainly in calves showing systemic signs, such as fever, depression, pneumonia, or septicemia. In this light, our results indicate that, in field practice, antimicrobial therapy was used very broadly, whereas intensive fluid therapy was implemented only rarely. This interpretation is reinforced by the data of Eibl et al. [38], who showed that 52.5% of respondents in four European countries declared using antimicrobials to treat neonatal calf diarrhea, 27% used them in every case, and 45% resorted to highest-priority critically important antimicrobials. At the same time, Bernal-Córdoba et al. [39] found that, despite screening 2899 publications, only 11 studies met the inclusion criteria for a systematic review, and the available evidence remains insufficient to evaluate the effectiveness of antimicrobials in the treatment of calf diarrhea.

The use of complete necropsy in combination with microbiological testing is fundamental to accurate classification of the cause of death. McConnel et al. [40] showed that agreement between diagnoses based on treatment records and postmortem classification was low, whereas agreement between necropsy findings and laboratory diagnosis was very high. Wilson et al. [18], in turn, showed that complete necropsy enables identification of the primary cause of death in 97% of cases and reveals important diseases that are often not correctly recognized ante mortem. For this reason, our material has considerable scientific value because it incorporates a broader diagnostic perspective rather than relying solely on clinical suspicion. Similar diagnostic challenges have been described elsewhere [27,41]. A limitation of our study, however, is that it included only calves referred for postmortem examination. The study provides a pathological and diagnostic characterization of fatal neonatal diseases based on systematic postmortem examinations rather than population-level mortality estimates. A further limitation is that the analyses did not account for within-herd clustering or shared exposure to management practices and pathogens.

## 5. Conclusions

In this retrospective necropsy-based study, diarrhea, particularly chronic and acute forms, and septicemia were the most frequently diagnosed causes of death among dairy calves submitted for postmortem examination during the first month of life. Diarrheal deaths occurred most often during winter and spring. Calves with septicemia were the youngest at death, whereas the highest age at death was observed in wasting cases. Among the factors analyzed, vaccination status showed the strongest association, with mortality being highest in acute diarrhea. By contrast, the highest mortality due to cachexia and septicemia was associated with colostrum management. Acute diarrheal mortality was associated mainly with ETEC, rotavirus, coronavirus, and *Cryptosporidium*. No statistically significant seasonality was demonstrated for the evaluated enteropathogens. Chronic diarrhea-related mortality, in turn, was more often associated with *C. parvum*. Treatment of calves was dominated by antimicrobial use, alongside insufficient application of fluid therapy, particularly in the most severe cases. The value of this dataset lies primarily in providing a detailed pathological and diagnostic characterization of fatal neonatal diseases in calves based on systematic postmortem examinations. Future studies should include prospective evaluation of the effectiveness of preventive programs and the use of risk-modeling approaches. There is also a need to develop specific treatment protocols tailored to different disease courses, with particular emphasis on fluid therapy.

## Figures and Tables

**Table 1:** Main Causes of Death in Dairy Calves within the First Month of Life (n = 498).

Cause of Death	n	%	Mean Age ± SD (days)
Chronic diarrhea	211	42.4	20.3 ± 5.6
Acute diarrhea	126	25.3	10.8 ± 5.2
Septicemia	54	10.8	7.7 ± 5.0
Cachexia/wasting syndrome	27	5.4	25.0 ± 3.7
Other causes	80	16.1	17.5 ± 7.8

Differences in age among diagnostic categories were assessed using the Kruskal–Wallis test (H = 226.39, df = 4, *p* < 0.001).

**Table 2:** Seasonal Distribution of the Main Causes of Death. Values are row %, (n).

Cause of Death	Winter ^1^	Spring ^1^	Summer ^1^	Autumn ^1^
Chronic diarrhea (n = 211)	31.3 (n = 66)	23.7 (n = 50)	17.1 (n = 36)	28.0 (n = 59)
Acute diarrhea (n = 126)	39.7 (n = 50)	37.3 (n = 47)	9.5 (n = 12)	13.5 (n = 17)
Septicemia (n = 54)	33.3 (n = 18)	18.5 (n = 10)	14.8 (n = 8)	33.3 (n = 18)
Cachexia/wasting syndrome (n = 27)	22.2 (n = 6)	29.6 (n = 8)	14.8 (n = 4)	33.3 (n = 9)
Other causes (n = 80)	28.8 (n = 23)	30.0 (n = 24)	13.8 (n = 11)	27.5 (n = 22)

^1^ Seasons were derived from calendar month: Winter (Dec–Feb), Spring (Mar–May), Summer (Jun–Aug), Autumn (Sep–Nov).

**Table 3:** Distribution of Causes of Death According to Farm Size.

Farm Size ^1^	Acute Diarrhea (%, n = 126)	Chronic Diarrhea (%, n = 211)	Septicemia (%, n = 54)	Cachexia/Wasting Syndrome (%, n = 27)	Other Causes (%, n = 80)
Large (≥200; n = 296)	26.4 (n = 78)	48.3 (n = 143)	7.1 (n = 21)	5.1 (n = 15)	13.2 (n = 39)
Medium (50–199; n = 119)	25.2 (n = 30)	27.7 (n = 33)	16.0 (n = 19)	9.2 (n = 11)	21.8 (n = 26)
Small (20–49; n = 83)	21.7 (n = 18)	42.2 (n = 35)	16.9 (n = 14)	1.2 (n = 1)	18.1 (n = 15)

^1^ Values are percentages within each farm-size group, with counts (n) in parentheses. The overall association was assessed using Pearson’s chi-square test (χ^2^ = 28.76, df = 8, *p* = 0.0003).

**Table 4:** Distribution of Recorded Calf- and Management-Related Characteristics across Diagnostic Categories ^1^.

Variable	Category	Chronic Diarrhea (%, n = 211)	Acute Diarrhea (%, n = 126)	Septicemia (%, n = 54)	Cachexia/Wasting Syndrome (%, n = 27)	Other Causes (%, n = 80)	χ^2^; *p*-Value
Vaccination	Vaccinated	69.7 (147)	34.9 (44)	35.2 (19)	51.9 (14)	45.0 (36)	χ^2^ = 48.81, *p* < 0.001
Vaccination	Non-vaccinated	30.3 (64)	65.1 (82)	64.8 (35)	48.1 (13)	55.0 (44)	
Colostrum	Proper	54.0 (114)	45.2 (57)	29.6 (16)	29.6 (8)	40.0 (32)	χ^2^ = 15.39, *p* = 0.004
Colostrum	Improper	46.0 (97)	54.8 (69)	70.4 (38)	70.4 (19)	60.0 (48)	
Access to water	None	4.3 (9)	7.1 (9)	5.6 (3)	0.0 (0)	7.5 (6)	χ^2^ = 16.58, *p* < 0.035
Access to water	Periodic	37.0 (78)	26.2 (33)	48.1 (26)	55.6 (15)	30.0 (24)	
Access to water	Adequate	58.8 (124)	66.7 (84)	46.3 (25)	44.4 (12)	62.5 (50)	

^1^ Values are percentages within each diagnostic category, with counts (n) in parentheses. The overall associations for vaccination status, colostrum management, and water access were assessed using Pearson’s chi-square test.

**Table 5:** Recorded pathogen detection in acute versus chronic diarrhea, with χ^2^, RR, OR, and 95% CI ^1^.

Pathogen	Acute Diarrhea (%, n = 126)	Chronic Diarrhea (%, n = 211)	χ^2^	RR ^1^	95% CI	OR ^1^	95% CI
*Salmonella* spp.	2.4% (3)	0.9% (2)	1.11	0.64	0.22–1.86	0.39	0.06–2.38
ETEC	51.6% (65)	9.5% (20)	74.17 **	0.31	0.21–0.46	0.10	0.06–0.18
Rotavirus	33.3% (42)	3.3% (7)	57.20 **	0.20	0.10–0.40	0.07	0.03–0.16
Coronavirus	23.0% (29)	0.5% (1)	49.43 **	0.05	0.01–0.34	0.02	0.00–0.12
*C. parvum*	25.4% (32)	37.4% (79)	5.18 *	1.22	1.04–1.43	1.76	1.08–2.87

^1^ RR and OR refer to chronic versus acute diarrhea among calves with recorded pathogen detection compared with calves without recorded detection of a given pathogen; Statistical significance at * *p* < 0.05; ** *p* < 0.01.

**Table 6:** Seasonal Distribution of Recorded Pathogen Detections in Diarrheal Cases (acute + chronic from Table 1). Values are row percentages, % (n).

Pathogen	Total n	Winter	Spring	Summer	Autumn	χ^2^; *p*-Value
*Salmonella* spp.	5	20.0 (n = 1)	60.0 (n = 3)	20.0 (n = 1)	0.0 (n = 0)	3.29; *p* < 0.349
ETEC	85	37.6 (n = 32)	27.1 (n = 23)	16.5 (n = 14)	18.8 (n = 16)	1.56; *p* < 0.669
Rotavirus	49	36.7 (n = 18)	26.5 (n = 13)	14.3 (n = 7)	22.4 (n = 11)	0.19; *p* < 0.979
Coronavirus	30	36.7 (n = 11)	43.3 (n = 13)	13.3 (n = 4)	6.7 (n = 2)	6.17; *p* < 0.103
*C. parvum*	111	32.4 (n = 36)	34.2 (n = 38)	13.5 (n = 15)	19.8 (n = 22)	2.51; *p* < 0.474

Seasonality was tested using Pearson’s chi-squared test on presence/absence by season in the combined diarrheal dataset classified as acute or chronic diarrhea in Table 1 (n = 337). Seasonal denominators were: winter n = 116, spring n = 97, summer n = 48, autumn n = 76.

**Table 7:** Supportive Fluid Therapy and Antibiotic Use by Cause of Death. Values are row percentages, % (n).

Cause of Death	No Fluid Therapy ^1^	Oral Rehydration Therapy	Intravenous/Subcutaneous Fluid Therapy	Antibiotic Therapy
Acute diarrhea (n = 126)	43.7 (n = 55)	50.0 (n = 63)	6.3 (n = 8)	89.7 (n = 113)
Chronic diarrhea (n = 211)	68.2 (n = 144)	27.0 (n = 57)	4.7 (n = 10)	87.7 (n = 185)
Septicemia (n = 54)	100.0 (n = 54)	0.0 (n = 0)	0.0 (n = 0)	85.2 (n = 46)
Cachexia/wasting syndrome (n = 27)	74.1 (n = 20)	25.9 (n = 7)	0.0 (n = 0)	81.5 (n = 22)
Other causes (n = 80)	85.0 (n = 68)	15.0 (n = 12)	0.0 (n = 0)	80.0 (n = 64)

^1^ Fluid therapy categories were coded as follows: 0—none, 1—oral, 2—intravenous, and 3—subcutaneous (categories 2 and 3 pooled). Antibiotic therapy was coded as yes/no.

## Data Availability

The raw data supporting the conclusions of this article will be made available by the authors on request.

## Supplementary Materials

The following supporting information can be downloaded at: https://www.mdpi.com/article/10.3390/ani16111743/s1, Supplementary Material File S1: Table S1.1. Questionnaire template: investigation of risk factors and causes of calf mortality; Table S1.2. Survey questions on management-related risk factors associated with cause-of-death categories in dairy calves during the first month of life; Table S1.3. Distribution of mixed enteropathogen detection patterns among calves with acute and chronic diarrhea; Table S1.4. Associations of supportive fluid therapy and antibiotic use with cause-of-death categories in dairy calves during the first month of life.
