# Supplementary material for: Necropsy-Based Analysis of Causes of Neonatal Mortality in Dairy Calves in Poland
Source: Animals (Basel). 2026 Jun 5;16(11):1743. doi: 10.3390/ani16111743 (PMC13255821; doi:10.3390/ani16111743)
Supplement: Supplementary file 1 [file animals-16-01743-s001.zip › animals-4291235-supplementary.pdf]

Supplementary material S1.

**Table S1.1.** Questionnaire template: investigation of risk factors and causes of calf mortality.

| Factor category | Item | Response options |
| --- | --- | --- |
| **I. General data** | Calf age | Number of days (1–30) |
| | Season | 1—Winter, 2—Spring, 3—Summer, 4—Autumn |
| | Farm size | S (small), M (medium), L (large) |
| **II. Prevention** | Dam vaccination | Yes (1) / No (0) |
| | Colostrum administration | Appropriate / Inappropriate |
| **III. Feeding** | Milk | Whole milk / Milk replacer |
| | Daily dose (min. 10–12% BW) | Yes (1) / No (0) |
| | Water access | 0—None, 1—Intermittent, 2—Continuous (adequate) |
| | Feeding method | Teat / Bucket / Tube |
| | Liquid feed temperature | Appropriate / Too low |
| | Technical errors | Incorrect powder concentration (1) / Improper preparation (2) |
| **IV. Treatment** | Antibiotic therapy | Yes (1) / No (0) |
| | Fluid therapy | 0—None, 1—Oral, 2—Intravenous (IV), 3—Subcutaneous (SC) |
| **V.Additional remarks** | | |

**Table S1.2.** Associations of management factor with cause-of-death categories in dairy calves during the first month of life.

**Vaccination: non-vaccinated vs vaccinated**

| **Diagnosis** | $\chi^2$ | **p** | **RR** | **95% CI** | **OR** | **95% CI** |
|---|---|---|---|---|---|---|
| Chronic diarrhea | 44.73 | <0.001 | 0.48 | 0.38–0.60 | 0.28 | 0.19–0.41 |
| Acute diarrhea | 20.21 | <0.001 | 2.04 | 1.48–2.81 | 2.58 | 1.70–3.93 |
| Septicemia | 7.03 | 0.008 | 2.01 | 1.18–3.42 | 2.19 | 1.21–3.94 |
| Cachexia/wasting syndrome | 0.00 | 0.970 | 1.01 | 0.49–2.11 | 1.02 | 0.47–2.21 |
| Other causes | 1.99 | 0.159 | 1.34 | 0.89–2.00 | 1.41 | 0.87–2.28 |

**Colostrum: improper vs proper**

| **Diagnosis** | $\chi^2$ | **p** | **RR** | **95% CI** | **OR** | **95% CI** |
|---|---|---|---|---|---|---|
| Chronic diarrhea | 10.53 | 0.001 | 0.71 | 0.58–0.88 | 0.55 | 0.39–0.79 |
| Acute diarrhea | 0.01 | 0.929 | 1.01 | 0.75–1.37 | 1.02 | 0.68–1.53 |
| Septicemia | 6.21 | 0.013 | 1.99 | 1.14–3.47 | 2.15 | 1.17–3.97 |
| Cachexia/wasting syndrome | 2.93 | 0.087 | 1.99 | 0.89–4.46 | 2.06 | 0.89–4.81 |
| Other causes | 1.20 | 0.274 | 1.26 | 0.83–1.90 | 1.31 | 0.81–2.13 |

**Access to water: periodic vs adequate**

| **Diagnosis** | $\chi^2$ | **p** | **RR** | **95% CI** | **OR** | **95% CI** |
|---|---|---|---|---|---|---|

| | | | | | | |
|---|---|---|---|---|---|---|
| Chronic diarrhea | 0.23 | 0.628 | 1.05 | 0.85–1.30 | 1.10 | 0.75–1.60 |
| Acute diarrhea | 5.58 | 0.018 | 0.66 | 0.46–0.94 | 0.58 | 0.37–0.91 |
| Septicemia | 4.53 | 0.033 | 1.74 | 1.04–2.92 | 1.87 | 1.04–3.36 |
| Cachexia/wasting syndrome | 4.05 | 0.044 | 2.10 | 1.00–4.37 | 2.20 | 1.00–4.81 |
| Other causes | 0.91 | 0.339 | 0.80 | 0.51–1.26 | 0.77 | 0.46–1.31 |
| **Access to water: none vs adequate** | | | | | | |
| **Diagnosis** | **$\chi^2$** | **p** | **RR** | **95% CI** | **OR** | **95% CI** |
| Chronic diarrhea | 0.77 | 0.380 | 0.79 | 0.46–1.37 | 0.69 | 0.30–1.59 |
| Acute diarrhea | 0.28 | 0.594 | 1.17 | 0.67–2.06 | 1.26 | 0.54–2.91 |
| Septicemia | 0.22 | 0.642 | 1.31 | 0.42–4.06 | 1.35 | 0.38–4.80 |
| Cachexia/wasting syndrome* | 1.14 | 0.286 | 0.42 | 0.03–6.95 | 0.41 | 0.02–7.16 |
| Other causes | 0.48 | 0.489 | 1.31 | 0.62–2.77 | 1.40 | 0.54–3.65 |

**Table S1.3.** Distribution of mixed enteropathogen detection patterns among calves with acute and chronic diarrhea.

| Mixed detection pattern | Acute diarrhea, n | Chronic diarrhea, n | Total, n |
|---|---|---|---|
| ETEC + rotavirus + bovine coronavirus + *C. parvum* | 12 | 0 | 12 |
| ETEC + rotavirus | 9 | 1 | 10 |
| ETEC + *C. parvum* | 4 | 5 | 9 |
| ETEC + bovine coronavirus | 4 | 0 | 4 |
| ETEC + rotavirus + *C. parvum* | 3 | 1 | 4 |
| ETEC + rotavirus + bovine coronavirus | 3 | 0 | 3 |
| Rotavirus + bovine coronavirus + *C. parvum* | 2 | 0 | 2 |
| Rotavirus + *C. parvum* | 0 | 2 | 2 |
| Rotavirus + bovine coronavirus | 1 | 0 | 1 |
| **Total mixed infections** | **38** | **9** | **47** |

Values are presented as numbers of calves. Only cases in which two or more enteropathogens were detected are included. ETEC, enterotoxigenic *Escherichia coli*; *C. parvum*.

**Table S1.4**. Associations of supportive fluid therapy and antibiotic use with cause-of-death categories in dairy calves during the first month of life.

**Oral rehydration therapy vs no fluid therapy**

| **Cause of death** | $\chi^2$ | **p** | **RR** | **95% CI** | **OR** | **95% CI** |
|---|---|---|---|---|---|---|
| Acute diarrhea | 45.40 | <0.001 | 2.81 | 2.08–3.81 | 4.31 | 2.77–6.70 |
| Chronic diarrhea | 0.06 | 0.806 | 0.97 | 0.77–1.23 | 0.95 | 0.64–1.42 |
| Septicemia* | 24.80 | <0.001 | 0.02 | 0.001–0.36 | 0.02 | 0.001–0.31 |
| Cachexia/wasting syndrome | 0.13 | 0.721 | 0.86 | 0.37–1.98 | 0.85 | 0.35–2.06 |
| Other causes | 9.09 | 0.003 | 0.43 | 0.24–0.77 | 0.38 | 0.20–0.73 |

**IV/SC fluid therapy vs no fluid therapy**

| **Cause of death** | $\chi^2$ | **p** | **RR** | **95% CI** | **OR** | **95% CI** |
|---|---|---|---|---|---|---|
| Acute diarrhea | 9.47 | 0.002 | 2.76 | 1.56–4.87 | 4.16 | 1.57–11.01 |
| Chronic diarrhea | 1.24 | 0.266 | 1.32 | 0.85–2.03 | 1.71 | 0.66–4.44 |
| Septicemia* | 3.36 | 0.067 | 0.17 | 0.011–2.57 | 0.14 | 0.008–2.40 |
| Cachexia/wasting syndrome* | 1.12 | 0.290 | 0.44 | 0.028–6.99 | 0.42 | 0.025–7.29 |
| Other causes* | 4.43 | 0.035 | 0.13 | 0.008–2.04 | 0.11 | 0.006–1.81 |

**Antibiotic therapy: yes vs no**

| **Cause of death** | $\chi^2$ | **p** | **RR** | **95% CI** | **OR** | **95% CI** |
|---|---|---|---|---|---|---|
| Acute diarrhea | 1.59 | 0.207 | 1.37 | 0.82–2.30 | 1.51 | 0.79–2.86 |
| Chronic diarrhea | 0.55 | 0.458 | 1.13 | 0.82–1.55 | 1.22 | 0.72–2.06 |
| Septicemia | 0.07 | 0.793 | 0.91 | 0.45–1.84 | 0.90 | 0.40–2.00 |
| Cachexia/wasting syndrome | 0.57 | 0.449 | 0.70 | 0.27–1.78 | 0.68 | 0.25–1.86 |
| Other causes | 3.25 | 0.071 | 0.63 | 0.39–1.03 | 0.57 | 0.31–1.06 |
